# Whole-system change: case study of factors facilitating early implementation of a primary health care reform in a South African province

**DOI:** 10.1186/s12913-014-0609-y

**Published:** 2014-11-29

**Authors:** Helen Schneider, Rene English, Hanani Tabana, Thesandree Padayachee, Marsha Orgill

**Affiliations:** School of Public Health, University of the Western Cape, Robert Sobukwe Road, Bellville, Cape Town 7535 South Africa; Health Systems Trust, Block B, Aintree Office Park, Doncaster Road Kenilworth, Cape Town, 7700 South Africa; Health Systems Trust, 34 Essex Terrace, Westville, Durban, 3630 South Africa; School of Public Health & Family Medicine, University of Cape Town, Anzio Road, Observatory, Cape Town, 7925 South Africa

**Keywords:** Whole-system change, Early implementation, Primary health care, Community health workers, South Africa

## Abstract

**Background:**

Whole-system interventions are those that entail system wide changes in goals, service delivery arrangements and relationships between actors, requiring approaches to implementation that go beyond projects or programmes.

**Methods:**

Drawing on concepts from complexity theory, this paper describes the catalysts to implementation of a whole-system intervention in the North West Province of South Africa. This province was an early adopter of a national primary health care (PHC) strategy that included the establishment of PHC outreach teams based on generalist community health workers. We interviewed a cross section of provincial actors, from senior to frontline, observed processes and reviewed secondary data, to construct a descriptive-explanatory case study of early implementation of the PHC outreach team strategy and the factors facilitating this in the province.

**Results:**

Implementation of the PHC outreach team strategy was characterised by the following features: 1) A favourable provincial context of a well established district and sub-district health system and long standing values in support of PHC; 2) The forging of a collective vision for the new strategy that built on prior history and values and that led to distributed leadership and ownership of the new policy; 3) An implementation strategy that ensured alignment of systems (information, human resources) and appropriate sequencing of activities (planning, training, piloting, household campaigns); 4) The privileging of ‘community dialogues’ and local manager participation in the early phases; 5) The establishment of special implementation structures: a PHC Task Team (chaired by a senior provincial manager) to enable feedback and ensure accountability, and an NGO partnership that provided flexible support for implementation.

**Conclusions:**

These features resonate with the deliberative, multi-level and context sensitive approaches described as the “simple rules” of successful PHC system change in other settings. Although implementation was not without tensions and weaknesses, particularly at the front-line of the PHC system, the case study highlights how a collective vision can facilitate commitment to and engagement with new policy in complex organisational environments. Successful adoption does not, however, guarantee sustained implementation at scale, and we consider the challenges to further implementation.

## Background

There is a global pendulum swing back towards support for community health workers (CHWs) in national health systems. The last few years have seen a growing literature on the potential roles and efficacy of CHWs in supporting progress towards the Millennium Development Goals [1] and calls for the “scaling up” of CHW programmes [2]. At the Third Global Forum on Human Resources for Health in Brazil in November 2013, key international players committed themselves to a framework for the strengthening and integration of CHW programmes within national health systems [3].

In 2010, South Africa introduced a policy of “primary health care (PHC) outreach teams” as part of a broader set of measures to revitalize the primary health care system [4]. The PHC outreach team strategy envisages that a team of generalist community health workers (CHWs), led and supported by a professional nurse, will be responsible for a defined number of households and will form close links with the local health facility. These teams will be comprehensive in orientation, addressing HIV/TB, maternal-child health and chronic non-communicable diseases, with a strong preventive/promotive focus that includes inter-sectoral action on the social determinants of health.

The PHC outreach team strategy in South Africa is less of a scale up than a re-organisation and integration of a loose and highly diverse community based “economy of care” [5] that has emerged in South Africa over the last 15 years around the response to HIV and TB [6]. With financial support from government and donors, an array of NGO-based, often single purpose, lay health workers were recruited to provide care, support and counseling in health facilities, homes and communities. In 2011, a government audit counted more than 72,000 such workers across the country, employed through 2,963 non-profit organizations (NPOs), many in a semi-formal relationship with the health system [7].

The development and implementation of PHC outreach teams, and CHW programmes more generally, can be regarded as policy with “system wide effects” [8], or what has also been variously referred to by Greenhalgh and colleagues as “whole-system change” [9], “whole-scale transformation” [10], or “large-system transformation” [11]. While not implying change in every single component of the health system, whole-system interventions are more than the implementation of a project or programme. They are *“aimed at coordinated, systemwide change affecting multiple organizations and care providers, with the goal of significant improvements in the efficiency of health care delivery, the quality of patient care, and population-level patient outcomes”* [11:422].

The PHC outreach team strategy entails new goals, forms of engagement with households, service delivery roles and relationships between players in the PHC system. Achieving such changes requires not only political support and the buy-in of a considerable number of actors, but also the mobilisation of new resources, development of new human resource and information systems, and significant changes in the orientations and everyday practices of frontline providers. It thus has planning, design, communication and political management elements that encompass both the “hardware” and “software” of health systems [12]. Together, these hardware and software elements form part of what the World Health Organization (WHO) refers to as the governance and leadership (or stewardship) roles of health systems [13]. In the past, numerous initiatives to scale up successful small-scale CHW models foundered because they failed to appreciate the governance and leadership tasks of both scaling up and sustaining such programmes at scale [14-16].

Because system wide interventions involve many players and sub-systems in complex webs of interaction (both formal and informal), the pathways and impacts of these interventions are inherently unpredictable. Complexity theory suggests, however, that most complex systems have a few key rules underpinning them [17]. In their review of Canadian experiences with primary health care reforms, Best et al. [11] identified five “simple rules” of successful large-system transformation.

They were:

1. A mix of designated (formal) leadership with distributed leadership in the change process
2. The presence of feedback loops
3. Paying attention to past system history
4. Engaging front line/powerful providers
5. Engaging end-users (families and communities)

The “simple rules” emphasize the need for collective or distributed leadership in the change process, and are therefore not only driven by top managers of organisations. Actors at the coal-face of systems, often the actual implementers of policy, may have very different interests and perspectives than their managers. Referred to as “street level bureaucrats” [18], they are faced with the immediate consequences – sometimes unanticipated - of new initiatives, and have to reconcile the demands from the top with the reality of resource constraints in the service delivery environment. They are able to exercise discretionary power in either accommodating or resisting policy initiatives and in shaping them in ways that fit with their every day realities. A political perspective on implementation, therefore, would see it as inherently contested and a negotiated combination of top down implementation with bottom up reactions and accommodations [19]. As implied by Best et al. [11] processes that explicitly seek to engage the frontline create the spaces for this negotiation.

In South Africa, the PHC outreach team strategy is a national policy whose implementation is managed in the nine provinces of the country. This paper is a case study of initial implementation in one of the early adopters of the new policy, North West Province. National stakeholders introduced the strategy in the Province in May 2011 at a workshop of more than 100 managers, spanning province to sub-district. The national Department of Health defined the roles and composition of the PHC Outreach Teams, developed training materials, provided a first round of training for the teams, and designed a new monitoring and evaluation system integrated into the district health information system. It stopped short, however, of providing financial resources for implementation of the new strategy. In this sense it could be regarded as a loose or unfunded mandate, and has resulted in highly variable uptake at provincial level.

The North West Province is one of the smaller and more rural South African provinces. It has a population of 3,6 million, an infrastructure of 22 hospitals and 300 clinics/community health centres and a well established district health system consisting of four health districts and nineteen sub-districts. Within weeks of the national visits, senior managers had set up a Provincial PHC Re-engineering Task Team to steer implementation. A provincial PHC Re-engineering Strategy and a three-year Project Implementation Plan followed soon afterwards, with all four districts subsequently integrating the strategy into their District Health Plans. Training of CHWs and Team Leaders began in late 2011 and 24 pilot teams were established, drawing on the existing pool of community based cadres and spanning all sub-districts. By mid-2012, more than 40,000 households had undergone an initial registration and screening visit and by the end of the year more than 90,000 follow up visits of households deemed vulnerable had been conducted. By mid-2013, 148 teams were in operation and by November 2013 more than 300,000 household visits had been recorded for the year. In 2012 and 2013, the reported household visits per capita were far higher in the North West than in any other province^a^. An audit in March 2014, counted 227 functioning teams, supported by 206 team leaders [20]. While it is too early to assess impacts of the intervention on coverage and public health indicators (such as early antenatal booking, immunization coverage and retention in care for chronic diseases), it is clear that the national policy gained ready anchor in this province and was able to mobilize change in a fairly short space of time.

In this paper we present the contextual factors and implementation strategies that enabled the uptake of the PHC outreach team strategy in the North West Province, and the lessons this offers for thinking about initiating change in primary health care systems and management of CHW programmes at scale.

## Methods

We report on an evaluation of the early implementation of the PHC outreach teams in the North West Province in the form of a qualitative, descriptive-explanatory case study [21]. As pointed out by Chen [22], early implementation represents a particular moment in the life of interventions, and assessments should therefore focus on aspects of change which are most relevant to this phase.

In this case study, we sought to establish a descriptive account of early implementation focusing on:

- Knowledge and ownership of the strategy amongst policy makers and managers
- The implementation strategy or “programme theory”^b^ adopted by the province and the chronology of actions which followed
- The mobilization of resources and system inputs (human resource, information, finance) for implementation
- Changes in the roles and relationships at the front-line of service provision (outreach teams, local facilities and their supervisors).

From this descriptive account and drawing on the notions of “simple rules” of change in complex systems, we then sought to identify, in the particular situational and historical context of the North West Province, catalysts for system wide change.

The project formed one of several case studies of early provincial implementation of PHC outreach team implementation, designed as rapid assessments providing a critical mirror on the unfolding policy process.

Data collection consisted of in-depth face-to-face and telephonic interviews and focus groups discussions with a wide range of actors across all levels of the health system. Respondents included those tasked with implementation (Task Team members and district “focal points”), senior line and support service managers (finance, human resource, other support services), middle managers and outreach team members. Interviews were conducted in all four districts. A total of 27 individual interviews and 9 focus group discussions were conducted (Table 1). The data collection processes/tools included open-ended narrative interviews, semi-structured interviews and structured checklist items. These were complemented by observations of meetings and a variety of documentary sources: conference and workshop presentations, speeches to parliament and project reports.

**Table 1:** **Study participants**

**Level**	**Participant**	**Individual interviews**	**Focus group discussions**
Senior management	Provincial PHC Task Team		1
	Provincial HR manager	2	
	District directors	2	-
Middle Management	Sub-district manager	1	-
	Sub- district PHC re-engineering focal person	3	-
Lower management	Facility managers	4	1
	Local area managers	6	-
Frontline providers	Outreach team leaders	9	1
	CHWs	-	6

The structured interviews and checklists were guided by the World Health Organization’s health system building blocks framework [13], outlining core functions such as governance & leadership, financing, information, supplies and human resources for health. These “hardware” elements of the health system were complemented by a focus on the more hidden “software” of implementation, such as actor knowledge and ownership of the policy, and changing roles and relationships.

Permission to conduct the study was granted by the North West Provincial PHC Re-engineering Task Team and the Provincial Health Research Committee, which actively assisted with setting up interviews with relevant players in each district/sub-district. Entry was certainly facilitated by the fact that two of our organizations (Health Systems Trust and University of the Western Cape) were in a partnership with the Province supporting PHC outreach team implementation at the time of the evaluation. However, the evaluation team was independent of the implementation team, and through processes of triangulation and reflexivity sought to minimise potential insider bias (whether positively or negatively inclined to the province).

All interviews were audio recorded, transcribed and coded. Interviews were divided up amongst the co-authors for analysis and then shared with others. Content analysis was conducted in an iterative process involving all the authors (in a series of meetings and workshops), first at a manifest level, to obtain a basic description of events and their sequencing and categorisation of data into main themes; and then at a deeper interpretive level to identify the key elements of the implementation ‘story’ in this province and what this might offer as lessons for elsewhere. A case study report was circulated and commented upon by provincial stakeholders and project partners, a key element of ensuring validity through member checking [21].

The University of Cape Town’s Ethics Committee granted clearance for the study. Written informed consent, including for recording of interviews, was obtained from each person prior to participation, and individual anonymity and the right to withdraw from the interview also guaranteed.

In the findings section which follows, we provide a descriptive account of early PHC outreach team implementation. In the discussion section we draw out explanatory themes.

## Results

### Knowledge and ownership by policy makers and managers

As already alluded to, commitment to the PHC Re-engineering policy (which also included a focus on school health services and specialist maternal-child health support) was evident at political and senior management levels. The provincial Minister of Health (referred to as the Member of the Executive Council or MEC) made central reference to PHC Re-engineering in his annual budget speeches^c^ and as indicated, senior executives moved rapidly to set up planning processes and structures to support implementation. Apart from the establishment of the PHC Re-engineering Task Team, one of the other first steps was to enter into a (donor funded) partnership with a national non-governmental organization (Health Systems Trust), which not only secured technical support for planning, but also skilled facilitators able to work with a range of internal and external actors. This partnership played a major role in supporting the implementation process: collecting and providing relevant information, designing the implementation strategy and engaging with front line providers.

There was also a high level of knowledge and ownership of the new policy by district and sub-district managers. In the North West Province, district managers are graded at a senior (Chief Director) level, and sub-district managers at Director level, above many managers in the provincial structures (a fact commented upon by provincial interviewees). They thus have status and decision making power. One of the four District Chief Directors chaired the PHC Re-engineering Task Team and, with the other district managers, became the key drivers of implementation. These managers understood their role as crucial:

*“At national I would say they would provide the policy but implementation is at district. The district shoots the force behind the implementation. If the districts are not participating in terms of the Chief Director and the Director myself it will be just a beautiful plan from national without being implemented.”* (Sub-district Manager)

They were able to achieve this on the back of long-standing and well-established district and sub-district management structures and systems of accountability. As one district manager explained:

*“Each district had structures that you would regard as sub-districts, which also had a manager and management team. We … looked into the issues of further management and local accountability and that’s when we appointed cadres known as health area managers where each sub-district was divided into smaller areas … for effective management and such areas were then managed by health area managers, while each facility had its own facility manager. That structure has been there before we adopted the PHC re-engineering.”* (District Director)

Planning and systems of reporting on the new policy thus became integrated into existing processes at district and sub-district level. The ready acceptance of the policy by district and sub-district managers was significantly influenced by the fact that they saw the policy, from the start, as both reflecting and reaffirming what was already present in the Province:

“*The elements of PHC re-engineering have long been implemented in the North West… The official adoption by the national department of the PHC re-engineering as a model upon which to push our service delivery perhaps has added … steam and focus in our province and almost confirms that what we have been doing is correct, and therefore strengthens what we were doing… It also affirmed the important role of community health care workers in service delivery. It therefore re-emphasized what we had started..*.” (District Manager)

This representation of the policy, as strengthening the existing ways of doing things in the province, surfaced in interviews at lower management levels as well and appeared to form an important part of driving implementation:

“*I see it as an integrated program and actually PHC re-engineering is revamping of the PHC that we have… As we go to pilot sites we tell them this is nothing new, we are just revamping that PHC we had.”* (Sub-district PHC Re-engineering Coordinator).

### Programme theory: Implementation structures and processes

A number of structures to support implementation were established. These structures included the PHC Re-engineering Task Team (hereafter referred to as the Task Team), secondment of a full time coordinator or “champion” from the provincial structures, and the appointment of coordinators at district and sub-district levels. These co-ordinators served as liaison between local area managers, team leaders and management in the district office and the province. They were represented on the Task Team, which met on a monthly basis and which formed the main communication channel up and down on implementation plans, progress and problems. The membership of the Task team included the four District Chief Directors (one of whom chaired the Task team), the Health Systems Trust (HST) and representatives of provincial Directorates for Strategic Programmes, District Health Systems, Hospitals, Finance, Monitoring and Evaluation, Communication and Human Resources.

From both observations and interviews, the Task Team was very effective and played a key role in sustaining momentum: minutes and action items were reviewed, and team members were expected to attend all meetings and report on a clearly defined set of milestones. These processes were replicated at district level, where PHC Re-engineering was a standing item on the agenda of the District Management Team meetings. As one sub-district manager commented:

*“There has never been a time we experienced a communication breakdown regarding issues of PHC re-engineering.”* (Sub-district manager).

These implementation structures were significantly bolstered by the partnership with the Health Systems Trust (HST). While in absolute terms the support HST provided was not large^d^, a flexible and attuned approach enabled it to address a range of needs, from conducting audits, to communication between levels, local coordination and facilitation and implementation of new systems. As one sub-district manager commented:

*“The government is a machine that operates very slowly, so you would find that the HST is way ahead in terms of interpreting what needs to be implemented on the ground.”* (Sub-district Manager)

HST’s engagement with frontline providers was commented on by a number of interviewees:

*“They [HST] don’t just end up at the district level they go down to the pilot sites speaking with the community health workers, assessing how they implement things, checking through their records.”* (Sub-District Manager)

The implementation process involved a sequence of steps starting with formal planning at provincial level. This was followed by a fairly intensive sub-district process, facilitated by HST, consisting of “community dialogues”, participatory planning, the nomination of CHWs and team leaders for training by district and sub-district players, the establishment of PHC outreach pilot teams (in each sub-district), and the mapping of households in pilot areas.

Combined with the orientation training provided by the national Department of Health, this local process effectively laid the ground for a reorganized and systematic approach to households and communities and changes in the work practices of CHWs. It started with an initial phase of registering and screening all households by the teams over a period of three months, with subsequent regular follow-up of “vulnerable” households (having a pregnant woman, children under the age of five years or someone receiving treatment for a chronic illness). Processes of screening and assessment followed structured formats provided by a nationally designed M&E and recording system.

The role of the PHC outreach pilot teams appeared to be less about demonstrating proof of concept than a methodology and a process to kick start implementation, with roll out built into the initial plans from the start.

Several interviewees highlighted the positive role of the “community dialogues”. Over the space of three months eighteen dialogues, in the form of participatory workshops, were held across the province. They were attended by a wide cross section of providers, community interests and institutions including:

- Traditional health practitioners
- Traditional Leaders
- Clinic Committees
- Home Based Carers
- Support group members
- Religious representatives
- Local government
- Department of Health
- Department of Policing and Community Policing Forums
- Department of Education
- Department of Labour
- Department of Social Development
- South African Social Security Agency
- Development Partners

The community dialogues served as mechanisms for information dissemination and mobilization of support for household profiling/registration as well as the new roles of CHWs. They were also reported through local radio stations. They became seen as a vital part of negotiating entry into communities and households:

*“The implementation dialogues must be carried out for the community to be aware of what is going to happen and they must accept because if they don’t that will cause us unnecessary challenges.”* (Outreach Team Leader)

Interestingly, they also established community participation and inter-sectoral action upfront as valued elements of the PHC outreach team strategy in the North West Province^e^. There was evidence that outreach teams participated in local inter-departmental “social cluster” (Education, Social Development etc.) structures, were able to table issues at ward (political) meetings, and in one ward had established successful relationships with the local Home Affairs Department.

### System inputs and resource mobilisation

By presenting the PHC outreach team policy as an extension or strengthening of core delivery functions, the province was able to mobilize the structures and resources of the district and sub-district health system, including the allocation of staff.

*“If it’s part of our mandate, then it’s in the equitable share [core budget]. It’s a good thing because we will own it 100% and we’ll plan and implement it accordingly.”* (Local area manager)

The involvement of the support cadres (human resources, finance, information) in the Task Team enabled the development of new systems such as more reliable payments of stipends, approval of transport and mobile phone allowances, and integration of the outreach team monitoring and evaluation system into the routine information system.

The Health Systems Trust played an important role in generating evidence for planning and implementation. It conducted a baseline audit of the numbers of CHWs in the province employed through non-governmental intermediaries and undertook geo-mapping of households in pilot wards to plan the household registration process. It also supported the implementation of the standardized M&E system in the province that included an mHealth pilot.

However, apart from these inputs, no additional financial resources were provided for implementation. A senior manager indicated that in the prevailing fiscal climate,

“*districts were being encouraged to “work differently” within the PHC re-engineering framework and obtain necessary budget accordingly.”*

Yet interviewees across the board saw the absence of dedicated financial resources from national or provincial government as the most significant threat to sustained implementation at scale. Funding was particularly required for the large numbers of additional nurses required as team leaders and to ensure a fairer remuneration dispensation for CHWs who were expected to work full time. Funding was also needed for vehicles for supervisory staff, supplies for the CHW kit bags, transport and cell phone allowances for CHWs, stationery, and filing cabinets for storing records. In reality, resources for implementation were being gradually mobilized from within district and provincial budgets and staff establishments, without the injection of additional external resources. Provincial managers were also actively considering alternative resourcing strategies:

*“[We should] train and use another cadre of health workers such as enrolled nurses. If we train them to be team leaders, or at least if we group wards so that one professional nurse supervises a number of wards, because we are running out of professional nurses.”* (District Director)

### Changing roles and relationships at the frontline

An audit conducted at the start of implementation identified a total 5,167 community based workers linked to the North West Provincial Department of Health, providing mainly home based care and DOTS (directly observed therapy) for TB, and 80% of whom had no accredited training. Seventy five percent were in receipt of government-funded stipends (R1,200 or US$120 per month), channeled through community-based organisations ^f^. Estimates in the planning phase were that about 3,500 CHWs (and +500 team leaders) would be required to achieve full coverage by PHC outreach teams in the province.

The PHC outreach team concept involved a significant reorganization of community-based services with changes in the roles, work practices and relationships amongst frontline players. CHWs were to provide comprehensive services (with induction in phases); they would be responsible for a defined number of households and take a population perspective, rather than care only for referred patients; their accountability would shift from NGO to local health facility and the formal health system and they would be actively supported by a team leader seconded from the local health facility. Finally, they were to engage with stakeholders outside the health sector.

A number of factors enabled this to happen, for the most part successfully, in the pilot sites of the North West Province. These were: 1) alignment of scopes of work, training and M&E systems (with, for example, structured processes of household screening), such that teams were able to begin their work with a sense of self-efficacy 2) community dialogues that facilitated entry in communities 3) the support and mediating roles of the team leader 4) and support and oversight from the sub-district/district management and the HST.

All the players interviewed – CHWs, team leaders, facility managers and their immediate supervisors (local area managers) – saw the value of the new approach. They believed it had brought tangible benefits in expanding access, improving performance of the health system (aspects such as vitamin A distribution, early antenatal booking, immunization coverage and retention in care of chronic cases all being mentioned), and building inter-sectoral relationships.

However, the implementation of the strategy also brought with it significant tensions. As a result many of the players at this level had an ambivalent attitude towards it. Chief among the tensions was the decision to recruit team leaders from within the nursing staff establishment of clinics. Facilities lost a staff member as the household registration was uncovering a large amount of unmet need and creating additional workloads. They were unprepared for this:

*“I was excited for our patients because the clinics are full. So they said they will be reducing the workload for us and the clinic will be empty as there will be nurses and community health workers who will be working outside the facility. [But] the members of staff are saying they are burdened with work because the sisters working on the PHC re-engineering have all the qualifications, but they are still referring to us at the facility”* (Facility Manager)

*“I was not aware that he [team leader] will be out of the facility permanently because I expected him to come back and to still allocate work to him.”* (Facility manager)

Team Leaders were faced with constant pressure to return to clinics:

*“We did our normal clinic work plus overseeing your community health care workers. …so we actually were responsible for two jobs at that time.”* (Team Leader)

Systems to reimburse team leaders for travel and communications were also not in place in all areas, and they had to *“pay from their pockets”* (Local area manager), even if eventually this was communicated and addressed through the PHC Task Team.

A second key problem was the frequently interrupted stipends of CHWs. Of the 36 CHWs interviewed in the group discussions, 20 of them had experienced some interruption in their stipend payments (for a mean of 3 months) since starting work as a lay health worker. In a subsequent visit to the province in 2013 following a period of unpaid CHW stipends, we noted that some teams were no longer functioning and professional nurses were back working in clinics. The non-payment of stipends was later resolved by moving the payment of all CHW stipends (which were simultaneously raised to R1,500 [US$150] a month) away from NGOs and onto the provincial payroll system. In the process, the NGOs who had previously deployed the CHWs for home-based care became sidelined by the re-organisation of services:

“*They [NGOs] were also furious to say we took their people, they developed them so much for home based care and we took them for our own benefit.”* (Local Area Manager)

In addition to these design or structural problems were those of process*.* The sheer number of players and turnover of staff meant that the intensive efforts at communication did not always reach the coal face. One team leader was recruited for training without any explanations and then expected to change her work without her consent:

“*We were just kind of taken out of our clinic situations, pushed into it and we had to do it… it’s very depressing to me because this is not really what I want to do.”* (Team Leader)

Because training was organised nationally, in some instances this was completed before facilities were prepared or community dialogues conducted. CHWs then showed up at facilities and facility managers who were not briefed *“didn’t know what to do*” (Facility Manager). They faced mistrust and resistance from households. Many local clinics also did not have the space to accommodate a whole new team of players, or a growing volume of paperwork, ultimately hampering their full integration in the workings of the facility. Many facility managers did not assume the envisaged oversight and support role to the teams and this shifted to the local area managers, who were under intense pressure from the sub-district structures to deliver on PHC Re-engineering.

Difficulties at facility level were compounded by occasional visits by the national Department of Health who made their own, sometimes contradictory demands on staff, and raised expectations. During one visit, in the initial stages of policy development national stakeholders made promises regarding the improved remuneration of CHWs:

“*One very big thing in this is, they [CHWs] were promised that they will get R3,000 [US$300] a month by national people. This was communicated in front of all the team leaders, and everybody that was present. Suddenly the new contract says R1,500 [US$150]. I mean, is that fair?”* (Team Leader).

Broken promises were a recurring theme in the interviews with CHWs, and despite overall support, also established an element of skepticism towards the strategy.

## Discussion

Table 2 summarises the strengths and weaknesses of the PHC outreach team implementation in the North West Province. As a formative evaluation exercise, these findings were fed back to, and discussed in, the provincial Task Team. Weaknesses such as the insufficient involvement of PHC facility managers were already understood and were being addressed. Some of the key constraints, such as fairer remuneration for CHWs, while well within the means of a middle income country such as South Africa, require policy and resource allocation shifts at national level. This did not, however, prevent the province from moving forward with a progressive roll out of the strategy.

**Table 2:** **Summary of strengths and weaknesses of implementation**

**Dimension**	**Strengths**	**Weaknesses**
Knowledge and ownership of the strategy	High level of knowledge and ownership amongst senior and middle level managers, and to a large extent, amongst CHWs and team leaders;	Primary health care clinic managers less well briefed and not fully owning the strategy;
Early implementation strategy	Establishment of PHC Task team and NGO partnership;	Sustaining intensive communication and engagement processes with local managers and communities in roll out phases;
	Alignment of systems (roles with training and M&E systems);	
	Appropriate sequencing of activities;	
	Community and local manager participation;	
Mobilization of resources and system inputs	Involvement of key directorates (HR, financing, information) in PHC Task team;	Team leaders appointed from existing staff establishments thus creating pressures on PHC facilities;
	Integration of strategy into district budgets;	No additional resources to ensure better CHW remuneration;
		Limited pool of professional staff to lead teams;
		Strains on local clinic infrastructure;
Changes in service delivery	Evidence of widespread adoption of new model;	Uneven integration into local PHC clinics.
	Supported by sub-district and local area managers.	

This case study also suggests a number of lessons for thinking about the catalysts for change in primary health care systems.

While *political and senior commitment* to policy, and the policy formulation, planning and design processes that follow, are necessary, they are not sufficient for ensuring implementation. In the North West Province, the *distributed ownership and leadership* of the policy by district and sub-district managers was central to the change process, which they drove through the Task Team and well-established district and sub-district structures. The commitment of these senior and middle managers emerged from a *collective vision* that framed the policy in continuity with the past and as reaffirming long held values in the province. Implementation was presented as a process of “revamping” and “strengthening” and not as something totally new. The task was explained to provincial stakeholders as a manageable one, which would be feasibly integrated into existing budgets and systems.

While the pre-existing district health system formed a ready *context* for implementation, the establishment of *special implementation structures* – the Task team and coordinators at various levels - and the *partnership* with the NGO - were key mechanisms for bringing together line and support managers, for up and down communication and problem solving, and for holding players *accountable*.

An *implementation process* that paid attention to system *inputs* (planning, information, workforce management, training, and their alignment), selection of *pilots* and a deliberate focus on *communication* and engagement with *communities* and local managers was also important. It also allowed for local involvement in decision-making (on for example choice of pilot sites). These communication and inclusion strategies were followed immediately by a *campaign* of household profiling, which showed visible commitment to implementation, whilst simultaneously identifying and responding to unmet needs.

These attributes of the implementation process follow closely the simple rules for primary health care system change proposed by Best et al. [11]. While strongly supported from the top, middle level actors emerged as powerful players in the process (designated with distributed leadership). They established the continuity of the policy process with the past (paying attention to system history). The Task Team provided a mechanism for identifying and resolving problems (feedback loops). Deliberative processes engaged end users and lower level managers and (to some extent) front line providers in participative decision-making.

Against this story of organisational and implementation strengths, however, is the difficult and more contested experience at the front-line of implementation. Implementation of the policy required not only changes in practices but also a shift in the allocation of resources, felt most acutely in local facilities and the NGOs from whom the CHWs were moved to the provincial system. Yet compared to other actors, facility managers appeared less informed and interactions with them were governed more by hierarchy and instruction, than consultation and collaboration. While supporting the new strategy, they also frequently expressed reservations, and sought to limit its impact on their own daily work and their participation in it. During the evaluation it became clear that that facility managers were a key cadre of health worker that should have been targeted with more direct communication on the new PHC strategy. While in this instance front line players (nursing professionals) may not have had the power of medical professionals in other systems to disrupt or prevent implementation, the case study highlights the crucial role of “street level bureaucrats” in policy processes and the discretionary spaces they exercise [18].

It appears that with time, however, there was adaptation to and acceptance of the new realities. Long standing and strongly held collective visions, the persistent efforts through the Task Team to resolve at least some of the problems and ongoing processes of accountability through sub-district and district structures, may have played some part in this.

The experience of the North West Province also has relevance for thinking about processes of scale up and governance, which, according to Liu et al. [23] have been inadequately theorized or documented in relation to CHW programmes. In their review of national CHW programmes in India, Pakistan and Ethiopia and Brazil, they point to the quality of background PHC systems as key to the strength and sustainability of CHW initiatives and further highlight the need for coordinated management across levels of the health system [23]. Both were key features of the North West Provincial case study. In contrast, a policy analysis of the establishment of the community health assistant cadre in Zambia, showed how inadequate attention to design and consultation and the privileging of powerful actors, including donors, produced a policy outcome that had low buy-in and limited chances of implementation [24].

## Conclusions

Against an apparently favourable backdrop, the “simple rules” of primary health system change described in other settings provided a plausible framing of how changes were achieved in the implementation of PHC outreach teams in the North West Province. They required attention to both the establishment of systems (finance, human resource, information etc.) and processes of deliberation and negotiation. In particular, the case study highlights how a collective vision can facilitate commitment to and engagement with new policy in complex organisational environments.

But if early processes powerfully set the stage and tone of further implementation, it is important to recognise that initiating whole-system change does not guarantee its sustainability over time. In the case of the North West Province, this will require persistence at all levels and an ongoing ability to learn, problem-solve, adapt and renew. At the time of writing, the project partnerships and implementation structures in the North West Province were still in place. If these were no longer in place, would implementation continue? Further evaluations would want to assess not only the extent to which processes such as training and information have become institutionalised in district systems, but also the degree to which front line actors and their supervisors are more knowledgeable and meaningfully engaged with outreach teams. As pointed out by numerous stakeholders, it is hard to envisage full coverage of the PHC outreach team strategy in the North West Province without additional resources (both people and funding). This will require a willingness to adapt elements of design (such as the team leader cadre) and mobilising additional resources for the strategy at national level.

Finally, the transferability of the “simple rules” identified in the North West Provincial case study to other contexts would need to be tested empirically. The notion of simple rules does, however, provide a way to consider the interactions between processes, contexts and interventions in designing and implementing health system reforms.

### Endnotes

^a^Extracted from routine DHIS data. In the other provinces, reported HH visits per population in 2013 varied from 10% to 65% of the North West Province levels.

^b^Defined as “stakeholders implicit and explicit assumptions on what actions are required to solve a problem” [22]

^c^See for example: North West gears up to improve primary health care http://www.sanews.gov.za/south-africa/north-west-gears-improve-primary-health-care

^d^The technical support included two full time equivalent staff with additional technical support for ad hoc activities: one staff member for three months to assist with community dialogues; two researchers for two months to complete CHW audit; one technical expert for one month to complete GIS mapping; six data capturers for two months to complete electronic capture of household profiles; and oversight/technical support from senior staff.

^e^As spelt out in a provincial PHC Re-engineering pamphlet: *“acceptance by the community and their participation in community-based health services is key to improving health outcomes.”*

^f^Jassat W. PHC Outreach Teams: North West Experience. Presentation to CHW Symposium, UWC, 7 June 2012.

## Abbreviations

CHW: Community health worker
DHS: District health system
HST: Health systems trust
NPO: Non-Profit Organisation
PHC: Primary health care
UWC: University of the Western Cape
